# Fetal weight estimation based on deep neural network: a retrospective observational study

**DOI:** 10.1186/s12884-023-05819-8

**Published:** 2023-08-02

**Authors:** Yifei Wang, Yi Shi, Chenjie Zhang, Kaizhen Su, Yixiao Hu, Lei Chen, Yanting Wu, Hefeng Huang

**Affiliations:** 1 International Peace Maternity and Child Health Hospital, Shanghai Jiao Tong University School of Medicine, Shanghai, 200030 China; 2 Shanghai Key Laboratory of Embryo Original Diseases, Shanghai, 200030 China; 3 Bio-X Institutes, Key Laboratory for the Genetics of Developmental and Neuropsychiatric Disorders, Shanghai Jiao Tong University, Shanghai, 200030 China; 4 Department of Mathematical Sciences, Tsinghua University, Beijing, 100084 China; 5 Obstetrics and Gynecology Hospital, Institute of Reproduction and Development, Fudan University, Shanghai, 200011 China; 6 Research Units of Embryo Original Diseases, Chinese Academy of Medical Sciences, , Shanghai, China; 7 Research Units of Embryo Original Diseases (No. 2019RU056), Chinese Academy of Medical Sciences, Shanghai, China

**Keywords:** Obstetrics and gynecology, Fetal monitoring, Fetal weight, Computer neural networks, Decision making

## Abstract

**Background:**

Improving the accuracy of estimated fetal weight (EFW) calculation can contribute to decision-making for obstetricians and decrease perinatal complications. This study aimed to develop a deep neural network (DNN) model for EFW based on obstetric electronic health records.

**Methods:**

This study retrospectively analyzed the electronic health records of pregnant women with live births delivery at the obstetrics department of International Peace Maternity & Child Health Hospital between January 2016 and December 2018. The DNN model was evaluated using Hadlock’s formula and multiple linear regression.

**Results:**

A total of 34824 live births (23922 primiparas) from 49896 pregnant women were analyzed. The root-mean-square error of DNN model was 189.64 g (95% CI 187.95 g—191.16 g), and the mean absolute percentage error was 5.79% (95%CI: 5.70%—5.81%), significantly lower compared to Hadlock’s formula (240.36 g and 6.46%, respectively). By combining with previously unreported factors, such as birth weight of prior pregnancies, a concise and effective DNN model was built based on only 10 parameters. Accuracy rate of a new model increased from 76.08% to 83.87%, with root-mean-square error of only 243.80 g.

**Conclusions:**

Proposed DNN model for EFW calculation is more accurate than previous approaches in this area and be adopted for better decision making related to fetal monitoring.

**Supplementary Information:**

The online version contains supplementary material available at 10.1186/s12884-023-05819-8.

## Background

The assessment of intrauterine fetal growth and development is an important issue in perinatal care. Estimated fetal weight (EFW) is a determinant of both maternal and fetal safety during pregnancy and delivery [1]. In the small-for-gestational-age (SGA) fetuses, EFW is an indicator of intrauterine growth retardation (IUGR) status and can determine delivery timing [2]. The EFW in the large-for-gestational-age (LGA) fetuses can potentially identify fetus macrosomia, which can lead to severe perinatal complications such as prolonged labor, fetal distress, shoulder dystocia and postpartum hemorrhage [1, 3]. Therefore, accurate EFW estimation before delivery is critical for determining the necessity of caesarean section and minimizing the risk of perinatal complications and mortality [4].

Although maternal anthropometrics based on the fundal height are easy to measure, this method can lead to a huge margin of error for the EFW [1], making EFW prediction unreliable. With widespread ultrasound (US) examination during various stages of pregnancy, formulae based on US measurements became the most widely used method of EFW [5]. The formulae derived from regression models using various combinations of US parameters, such as Hadlock’s formula [6] and Warsof’s formula [7], were well established but still have 6.5–8% mean absolute percentage error (MAPE) [8]. Although formulae based on fetal thigh or upper arm volume measurements through 3-D US [9] and magnetic resonance echo-planar imaging [10] can predict EFW with higher accuracy, these approaches are time-consuming, expensive and technically challenging.

Artificial neural network (ANN) is a computational analog of a biologic neural system. ANN is a non-linear, self-learning system composed of numerous independent processing units [11], and can therefore process a large amount of data simultaneously [12]. In recent years, deep neural network (DNN) models made significant achievements in numerous computational biology and medicine problems [13, 14]. In addition, several research groups have utilized ANN to construct prediction models for EFW. Farmer et al. first used the neural network method to obtain EFW of LGA fetus, and predicted the EFW of 100 potential LGA fetuses with a MAPE of 4.7% compared to 10% achieved by conventional methods [15]. Chuang et al. devised an ANN model for estimating birth weight, which demonstrated a MAPE of only 6.15% as opposed to 7.5% by the conventional approach [16]. These methods do not require an empirical formula, and can easily adapt to more parameters that are potentially related to EFW. However, no prediction model has so far been used in clinical practice.

Therefore, this study aims to develop a clinically applicable model for EFW prediction based on DNN.

## Methods

### Study design and population

This study retrospectively analyzed the electronic health records of pregnant women with live births delivery at the obstetrics department of International Peace Maternity & Child Health Hospital between January 2016 and December 2018.

**Fig. 1:**
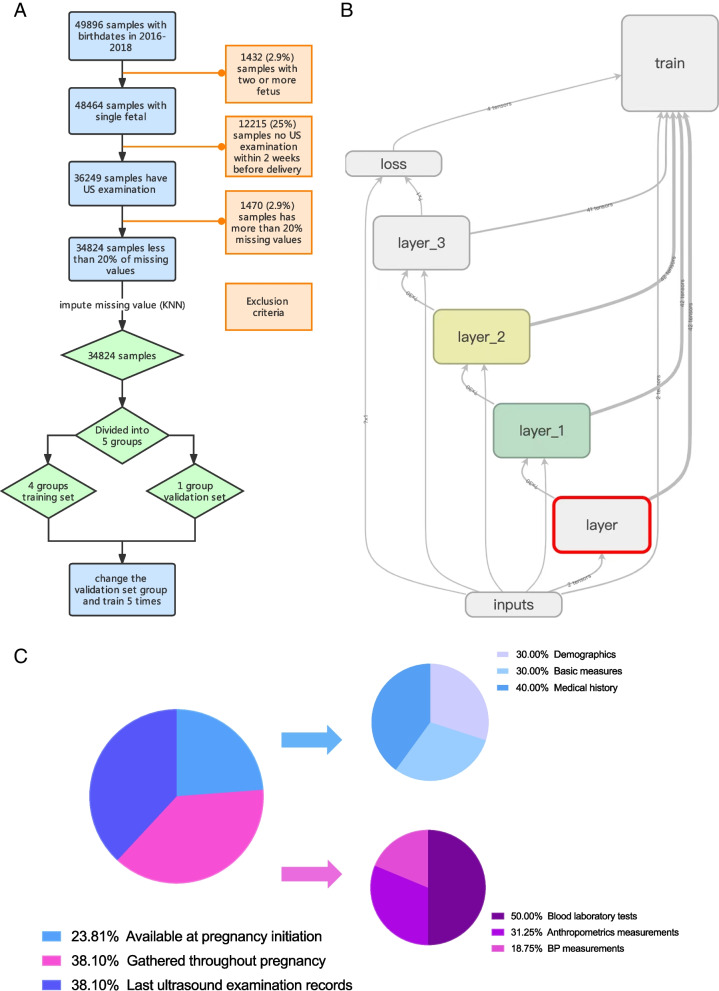
The flow chart of cohort selection, feature distribution and Deep Neural Network (DNN) model. **A** Cohort selection. Pregnancies were first identified by offspring birth date. Cases with anomalous fetuses, multifetal gestations and without Ultrasound examination within 2 weeks before delivery were excluded. Finally, the cohort was divided into training and validation sets (see Methods). **B** DNN model. **C** Feature availability distribution. Pie charts are divided according to the sum of features in each feature set

The inclusion criteria were as follows: pregnancy, age 18–49 years, gestational week 28–41 + 6 weeks, US examination within 14 days before delivery. We excluded the features with too much missing data (more than 20% missing data), coefficient of variation (C.V%) < 2%, or difficult to calculate (such as multicategorical features). The remaining 42 parameters were included for further analysis and screening. Cases of anomalous fetuses, with more than 20% missing data, or with illogical values for the US parameters were excluded. For cases with < 20% missing values rate, the missing values and illogical values were input via K-nearest neighbor (KNN) imputation of *sklearn* [17] (k = 5 by default) (Fig. 1A).

This study was approved by the Medical Ethical Committee of International Peace Maternity and Child Health Hospital, School of Medicine, Shanghai Jiao Tong University (No. GKLW2020-01). This study have obtained both informed consent and ethics committee approval for studies on patient records. The unified ID of unknown personal information was used to protect the privacy of the patients.

### Data collection

A dataset of 42 obstetric features was constructed, of which 10 features were available at the initiation of pregnancy, 16 were obtained during the pregnancy and 16 from the last US examination records. The initial obstetric features included demographics (e.g. age, parity, gravidity), basic measures (e.g. weight and height), and medical history of the current pregnancy (e.g. previous neonatal weight, IVF etc.). The features gathered during pregnancy included blood laboratory tests (e.g. OGTT, triglyceride, cholesterol), anthropometrics measurements (e.g. fundal height, weight gain) and BP measurements. All data were collected during antenatal visits or admission prior to delivery, and there was no selection bias. US examination records was based on a standard US template, which have amniotic fluid depth of 4 quadrant and a total AFI, abdominal transverse diameter, abdominal anteroposterior diameter, but no abdominal circumference. All features and their mean values are listed in Fig. 1c, and the percentage of feature availability per category is shown in Fig. 1B.

### Statistical analyses

Statistical analyses were performed with Python 3.7.2 scikit-learn 0.21.3, Keras 2.2.5, Shap 0.35.0, IBM SPSS 25 for mac (IBM Corp, Armonk, NY) and R 4.0.0.

#### DNN model building

**Fig. 2:**
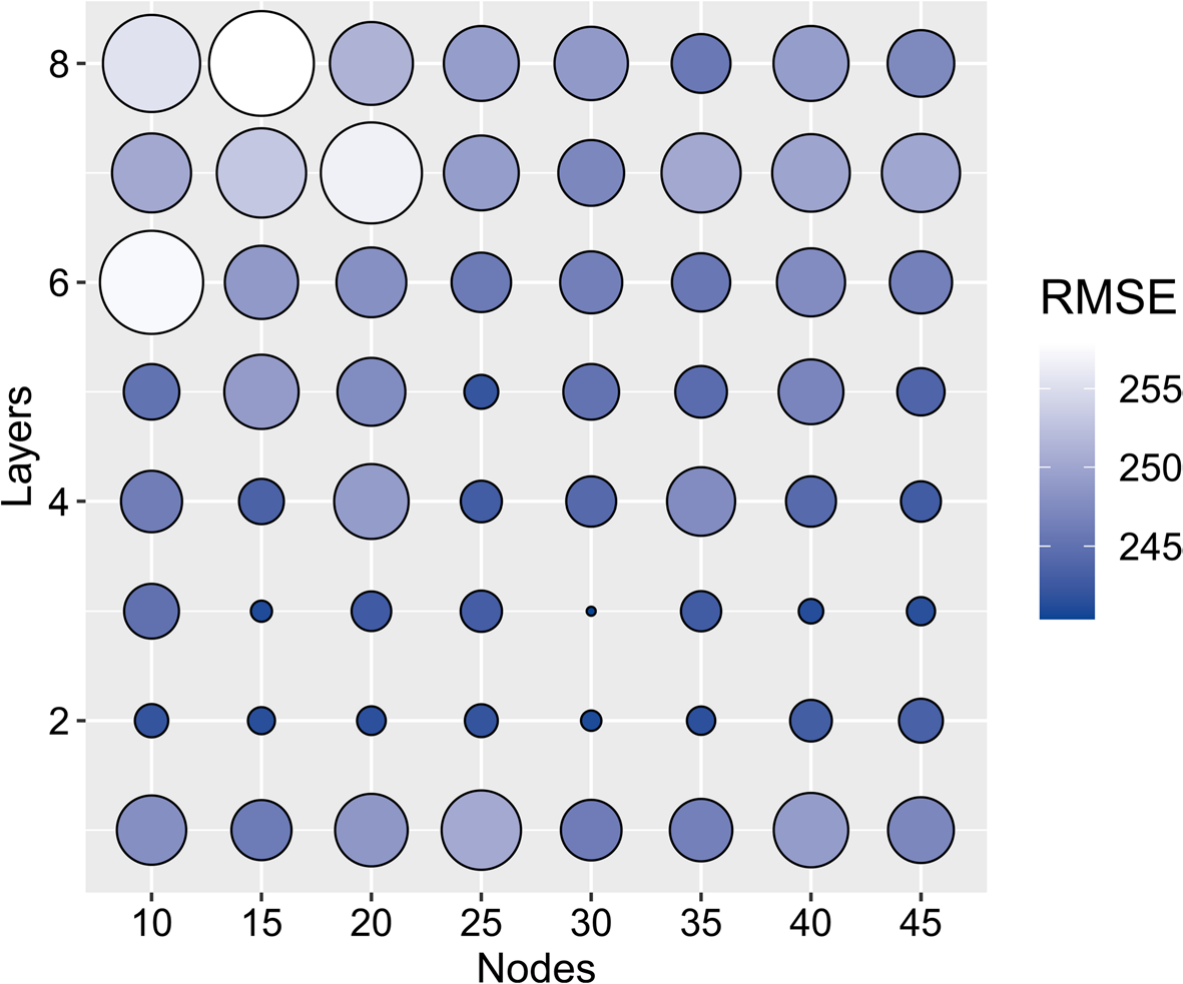
The change in Root Mean Square Error (RMSE) across different layers and nodes during pre-training. The size and the color of the bubbles both show RMSE

The DNN model was built via Tensorflow 1.13.1. It consisted of the input layer (all input features), the output layer (EFW) and several hidden layers between them. Every two adjacent layers were fully connected. The number of layers and nodes in the hidden layers were determined according to the average results from fivefold cross-validation (Fig. 2), which had 3 hidden layers with 30 nodes per layer. Considering that the input feature space has been limited with no salient prior knowledge to be abstracted, convolution layers were not used before the fully connected layers of the DNN. In order to avoid the cumulative effect of uneven distribution and to increase the network converge, batch normalization was used between every two layers.

Except for the output layer, the excitation functions between each pair of layers were *ELU* [1, 18]:

1 $$\begin{array}{cc}\mathrm{ELU}\left(x\right)= {\{}_{\alpha \left({e}^{x}-1\right)}^{x}& \begin{array}{c}\mathrm{if}\quad x>0\\ \mathrm{if} \quad x<0\end{array}\end{array}$$

The loss function (cost) is mainly based on the mean-square error (MSE), supplemented with the L1 and L2 regularization coefficients (λ = 0.0001) of the weights of each layer to avoid model overfitting.

#### DNN model training

The back propagation network algorithm was used as the learning algorithm to train the DNN, The Adadelta algorithm provides an adaptive learning rate as a training optimizer [19]. The number of training epochs is also adaptive. The loss of the training dataset was calculated at each epoch, and if the loss_i_ of the training set in 1000 consecutive epochs was greater than the minimum loss of the verification set (loss_min_), the iteration process was terminated, and the network with the smallest loss was selected as the final model. The robustness of the machine learning model was validated by the fivefold cross and dropout methods. Before training, the remaining data was randomly divided into 5 groups, with one group as the validation dataset, and the remaining 4 combinations as the training set. A dropout (fraction = 0.2) step between layers was used to prevent overfitting and increase the generalization of the model. The flow chart for the development of DNN model is shown in Fig. 1B.

#### Model interpretations

The contribution of individual features to the model output was determined in terms of Shapley values [20] and their related extensions using the SHAP (SHapley Additive exPlanations) package [21], which partitioned the prediction result of every sample into the contribution of each constituent feature value. The average SHAP values of each feature across all samples were calculated to estimate the individual contributions.

#### Feature selections

Feature selection was performed to reduce the data dimension (avoiding potential overfitting) as much as possible without affecting the accuracy of training, and obtain a small panel of features while ensuring the generalization and adaptability of the training results. The model-free Greedy (MFG) algorithm was used since it does not rely on a specific model and can intuitively select a set of features with the highest estimation/classification efficiency [22]. Since the MFG approach depends on various correlations, it has performed optimally in previous machine learning applications [22].

#### Methods comparison

The performance of the DNN model was evaluated using Hadlock’s formula and multiple linear regression (MLR) as the reference. The EFW was calculated by Hadlock’s formula using US parameters. For the MLR model, the research parameters were screened in a step-by-step manner, and those with *p* < 0.05 were included and those with p > 0.1 were excluded. The confidence interval of MAE, MAPE and RMSE were calculated by SPSS.

#### The evaluation methods of different models

(1) Mean percentage error (MPE): percentage of the error between the EFW and the actual birth weight of each individual was calculated, with following calculation of the mean value and its standard deviation. (2) Mean absolute percentage error (MAPE): percentage of the error between the EFW and the actual birth weight was calculated, after that the absolute value was taken to calculate the average value and standard deviation. (3) Mean absolute error (MAE): absolute error between the EFW and the actual birth weight was directly calculated and then averaged.

(4) Root mean square error (RMSE): the standard deviation of the error between the EFW and the actual birth weight, as commonly used in regression analysis. (5) Prediction accuracy rate: the accuracy was defined as the predictions error < 10% of actual birth weight. According to the traditional standard of obstetrics, the difference between the predicted weight and the actual birth weight less than 250 g is regarded as an accurate prediction, and the accuracy rate is calculated by dividing the accurately predicted sample by the population.

## Results

A total of 34814 live births (23922 primiparas and 11422 multiparas) from 49896 pregnant women were analyzed (Fig. 1A). The majority (97%) of pregnant women were of Han Chinese ethnicity. The MLR and DNN models were first constructed using all 39 features (Fig. 1B-C; Table S1). The models were then pre-trained and the optimal number of layers and the number of nodes in each layer were obtained by cross-validation. The changes in the RMSE of each network during pre-training are shown in Fig. 2 and Table S2. The final selected network consisted of 3 hidden layers with 30 nodes per layer, which has least RMSE.

**Fig. 3:**
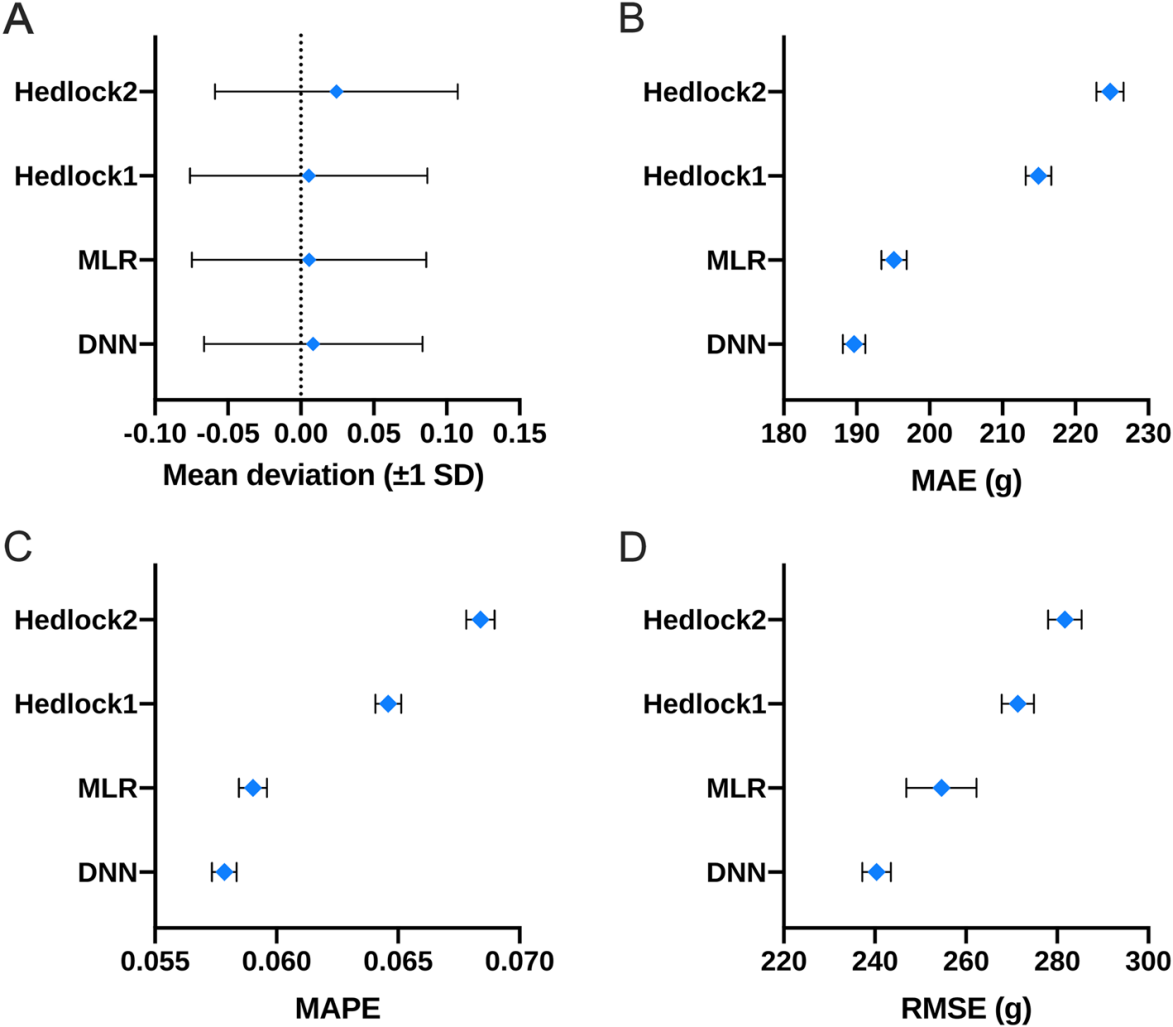
The performance of different methods for estimated fetal weight (EFW). The mean deviation (± 1 SD). **A** mean absolute error (MAE), (**B**) mean absolute percentage error (MAPE), (**C**) and root-mean-square error (RMSE), (**D**) and their 95% confidence interval of EFW in different methods

**Fig. 4:**
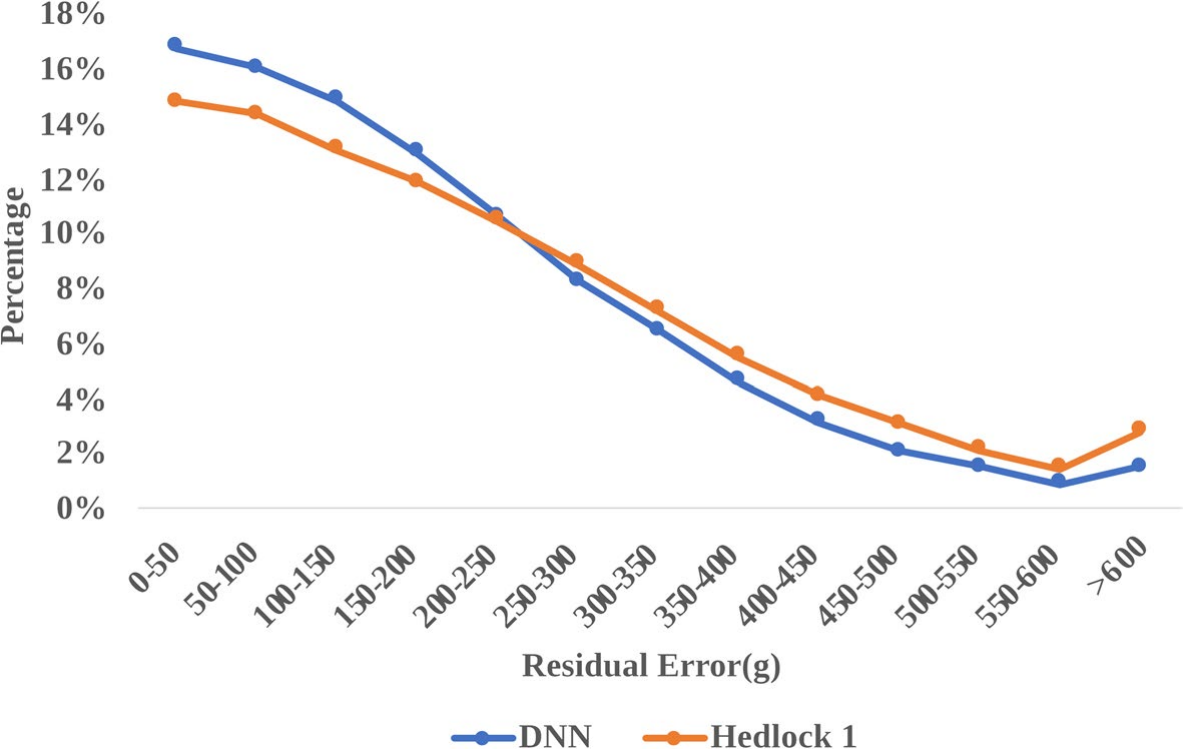
The frequency distribution of the estimated error of the Deep Neural Network (DNN) model and the Hadlock’s formula in the calculation of estimated fetal weight

**Fig. 5:**
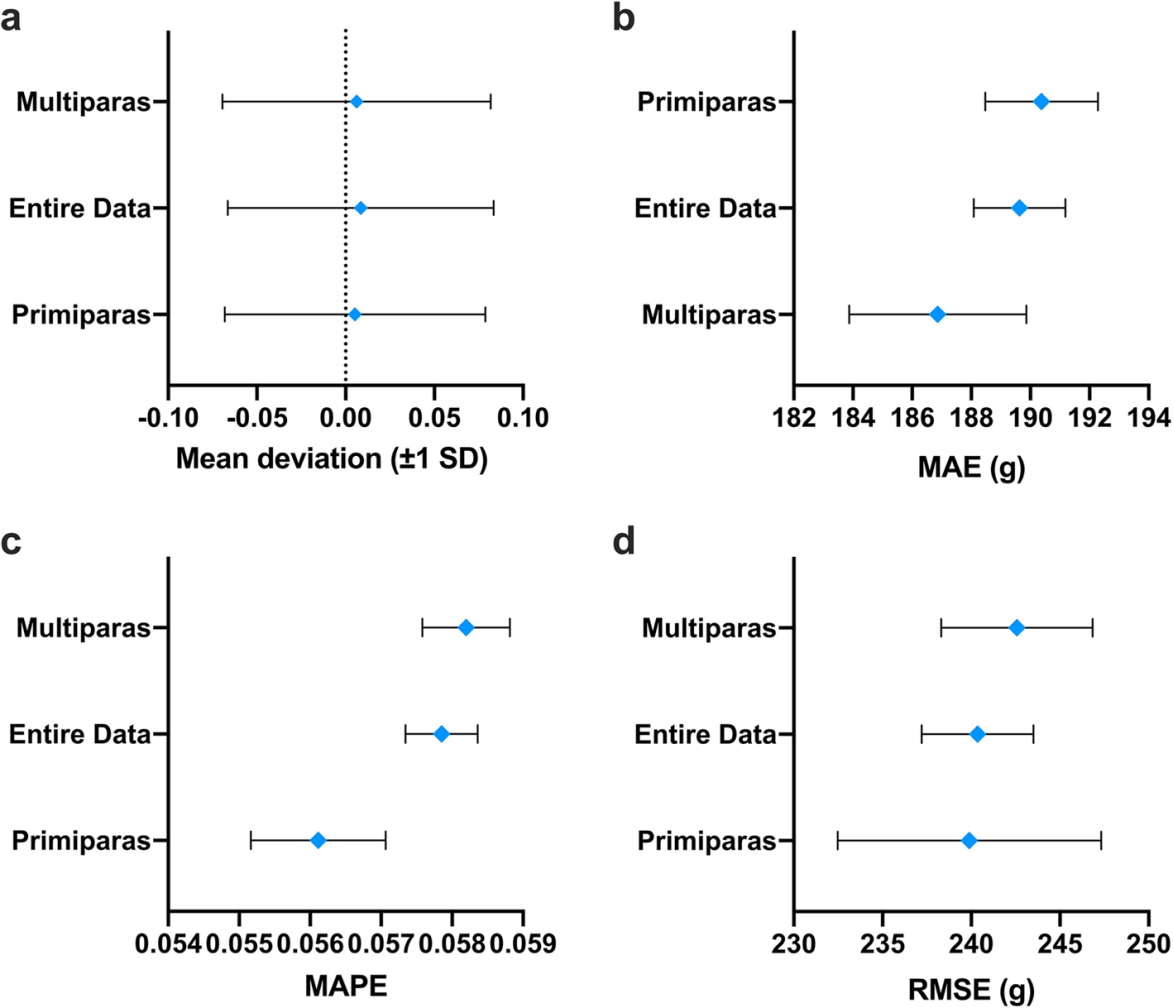
The performance of different methods in the primiparas and multiparas groups. The mean deviation (± 1 SD) (**A**) mean absolute error (MAE), (**B**) mean absolute percentage error (MAPE), (**C**) and root-mean-square error (RMSE), (**D**) and their 95% confidence interval of the estimated fetal weight (EFW) in the primiparas and multiparas groups compare to the entire cohort

The MAE, MAPE and RMSE of the EFW on the validation dataset were calculated by Hadlock’s formula, MLR model and DNN model. The DNN model achieved MAE of 189.64 g (95% CI 187.95 g—191.16 g) compared to 214.95 g (95% CI 213.48 g—217.10 g) by the Hadlock’s formula. In addition, the MAPE of the DNN model was only 5.79% (95% CI 5.70%—5.81%) compared to 6.46% (95% CI 6.41%—6.52%) achieved by Hadlock’s formula. Finally, the RMSE achieved by DNN and Hadlock’s method were 240.36 g (95% CI 238.24 g—242.31 g) and 271.35 g (95% CI 269.69 g—274.15 g) respectively (Fig. 3 A-D, Table S3). Accordingly, the accuracy rate of EFW increased from 76.08% with the Hadlock’s formula to 83.87% using the DNN model, corresponding to an increment of 7.79% (Table S3). There were also significant differences between the DNN model and the MLR method (Table S3). Furthermore, the frequency of samples with large deviation (> 500 g) was only 3.95% in the DNN model compared to 6.45% with the Hadlock’s formula, indicating that the DNN model can significantly reduce estimated errors (Fig. 4). The predictive accuracy of DNN was also tested on primiparas and multiparas (previous neonates data available). As shown in Fig. 5, the DNN model had lower mean deviation, MAE, MAPE and RMSE compared to the Hadlock’s formula in both subgroups, and the predictive performance of DNN was better for the multiparas group.

**Fig. 6:**
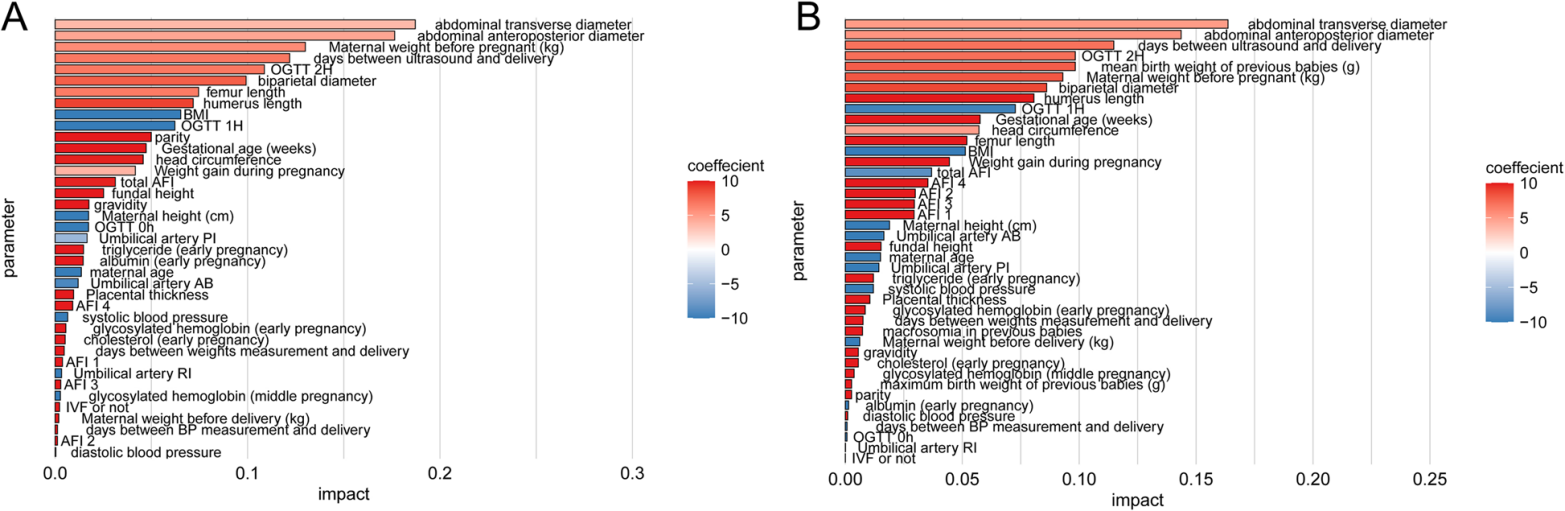
Feature impact of all contributing features. **A** The entire data model. **B** Multiparas model. Bar colors indicate direction of influence based on the correlation coefficient of a feature

**Fig. 7:**
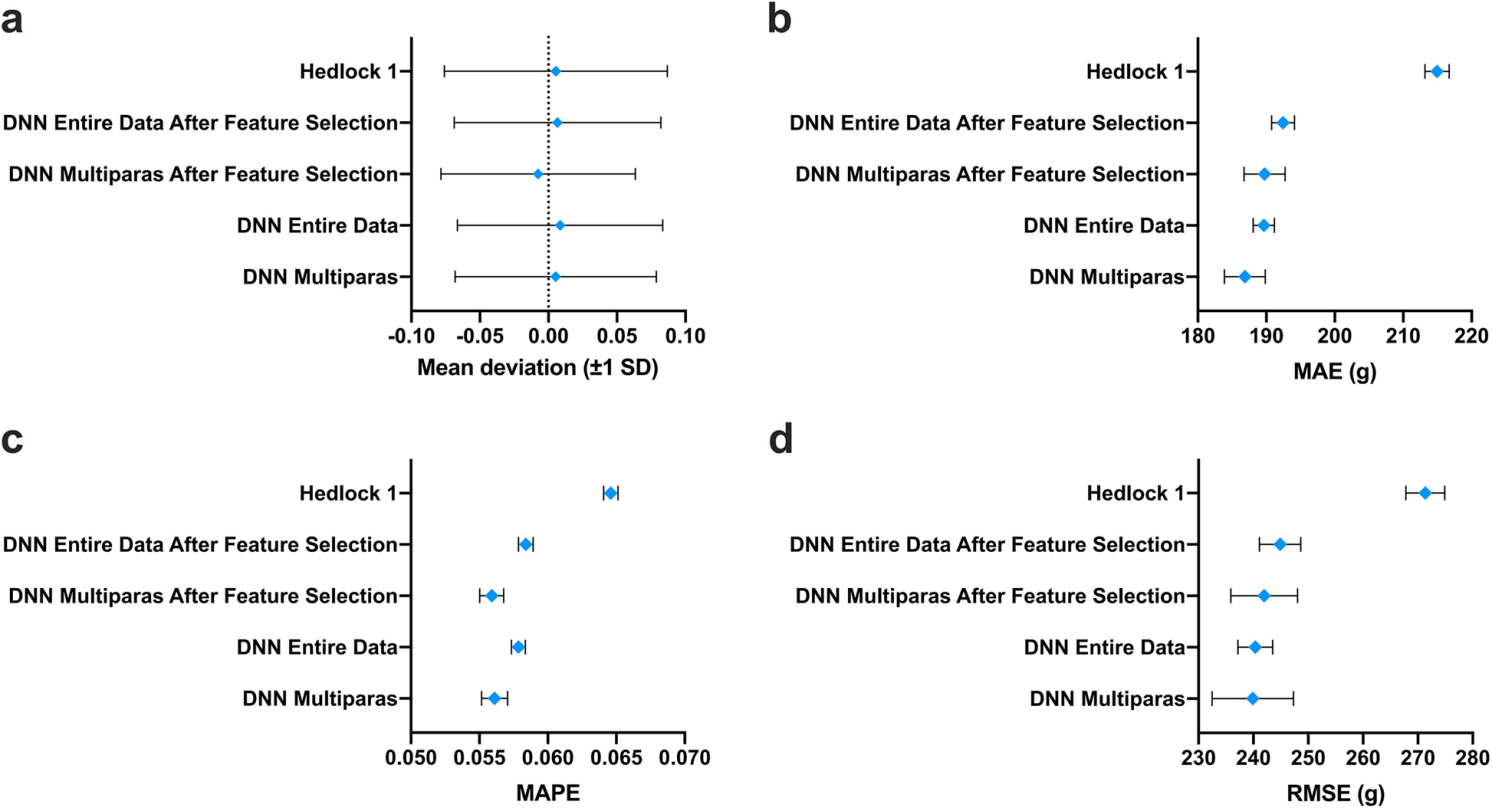
**A**-**D** The performance of models after feature selection in the primiparas and multiparas groups and the entire cohort. The mean deviation (± 1 SD), (**A**) mean absolute error (MAE), (**B**) mean absolute percentage error (MAPE), (**C**) and root-mean-square error (RMSE), (**D**) and their 95% confidence interval of the EFW of multiparas group and the entire cohort after model-free Greedy (MFG) algorythm feature selection versus Hadlock’s formula and the all features model

**Fig. 8:**
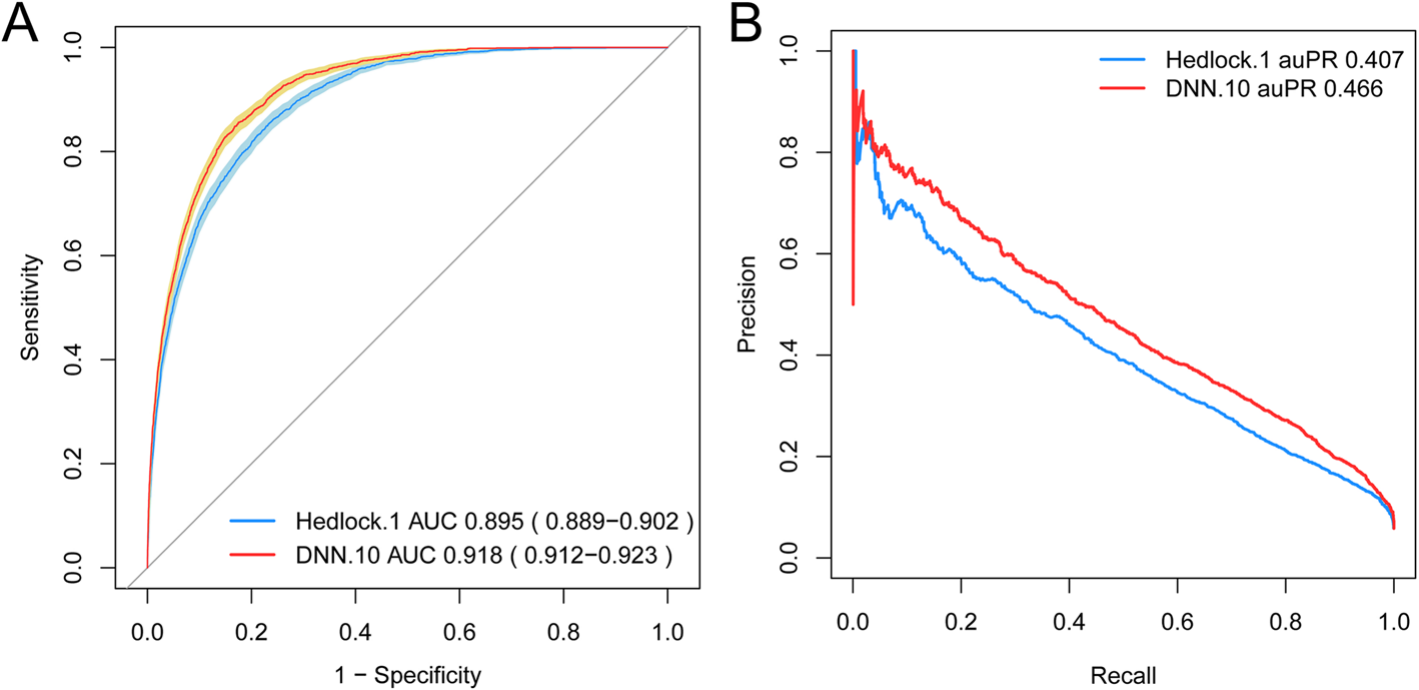
The receiver operating characteristic (ROC) and precision-recall curve (PR) curve of Deep Neural Network (DNN) model after feature selection versus Hadlock’s formula on macrosomia prediction. **A** ROC curve. **B** PR curve

Deep LIFT analysis showed that apart from the US data, the most predictive feature for EFW was pre-pregnancy maternal weight (kg), followed by the maternal OGTT 2H and BMI (Fig. 6A). In the multiparas group, the most predictive feature for EFW was OGTT 2H, followed by mean birth weight of previous babies (g) and pre-pregnancy maternal weight (Fig. 6B). As shown in Table S4, we selected 10 features, of which 6 were US data. The simpler model was trained on the entire data set and achieved MAE of 192.21 g (95% CI 190.54—193.87), MAPE of 5.81% (95% CI 5.75%—5.86%) and RMSE of 243.80 g (95% CI 241.66—245.91), all of which were superior to that of Hadlock’s formula (Fig. 7, Table S5). To further evaluate its performance in the multiparas group, we added the previous neonatal birth weight feature. As expected, the concise DNN model performed better for multiparas cases, thus confirming that inclusion of previous neonatal birth weight improves the accuracy of EFW (Fig. 7, Table S6). Finally, we evaluated the ability of the DNN model after feature selection to predict macrosomia (neonatal weight > 4000 g). The DNN model achieved an AUROC of 0.918 and area under the precision-recall curve (AUPR) of 0.466, compared to 0.895 and 0.407 respectively by the Hadlock’s formula (Fig. 8, Table S7).

## Discussion

### Main findings

This study showed that the root-mean-square error, as well as the mean absolute percentage error of DNN model were significantly lower compared to Hadlock’s formula. After considering previously unreported factors, such as birth weight of previous babies, new concise and effective DNN model was proposed, with accuracy rate increased from 76.08% to 83.87% and root-mean-square error of only 3.44 g. Taken together, proposed prediction model was more accurate compared to both Hadlock’s method and MLR in predicting the EFW and therefore have a potential for aiding decision making in fetal development monitoring.

### Strengths and limitations

In recent years several EFW prediction models have been developed, but none of these models were practiced in routine clinical applications or recommended according to the current guidelines. Hadlock’s formula is still thus far one of the best-established weight estimation formulas and the one most widely used in everyday routine work, despite recent studies indicating potential ways to decrease MAPE, the especially in macrosomic fetuses and other deviant cases [8, 23]. Although, due to the drastic difference in the number of participants, the MAPE in this study was slightly higher than that calculated by Konwar [8] or Weiss [23] (5.79% for DNN estimation and 6.46% for Hadlock’s formula), the DNN model still predicted the EFW more accurately compared to the Hadlock’s formula. Moreover, the frequency of samples with large deviation (> 500 g) was also significantly smaller compared to Hadlock’s formula, indicating that the DNN model can reduce large estimated errors.

One of the major issues regarding EFW is fetal development monitoring, and the currently used Hadlock’s formula have previously demonstrated notably worse performance in SGA and LGA groups [3, 23]. Based on the auROC values, we propose that the 10-feature DNN model can make 1006 more accurate weight predictions and 189 fewer missed diagnosis of macrosomic babies at our hospital, suggesting it can help doctors make more reasonable decisions regarding the necessity of caesarean sections, which might reduce shoulder dystocia [24]. Our results suggest that the DNN prediction model can track fetal weight easily and accurately during pregnancy and find SGA/LGA fetus earlier, which can help determining whether intervention is necessary.

Other than well-known parameters of EFW such as US data and maternal weight gain, our selection method revealed predictive factors that were not previously reported, especially in the multiparas group. The main newly identified parameters were main and maximum birth weight of previous pregnancies in multiparas group. While women with a history of fetal macrosomia and SGA in previous pregnancies were shown to possess the increased risk of the same in their current pregnancy [25–27], we found that the birth weight of previous babies was notably more predictive of EFW than previously reported. Although maximal prediction accuracy requires the entire EHR, we demonstrated that 10 features selected by the MFG method, which can be easily collected by questionnaires and physical examinations, can still achieve accurate prediction. This also presents the possibility of accurate EFW via web- or smartphone-based self-assessment tools due to its light computational complexity.

There are several limitations in our study that ought to be considered. Firstly, our prediction model was based on retrospective EHR data of one center that have inherent biases and were influenced by patients’ interactions. However, these biases were reduced to an extent since the outcome of the model was based on routine pregnancy tests that were comprehensively documented in the EHRs. Secondly, we were not able to continuously train the model with more data. Although our hospital is one of the three major maternity hospitals in Shanghai, serving more than 10,000 pregnant women all over Shanghai and surrounding areas every year. We agree that the dataset may still not represent the population of pregnant women in other regions, especially for women outside of Shanghai. Larger cohorts and multi-center validation are needed to further decrease the biases. Thirdly, we only had one doctor to access the US measurements, which were not validated by a second independent investigator. Fourthly, although we use the interval between US examination and delivery as a feature to adjust the EFW model, the fetal growth during the last few days can still lead to large random errors. Further studies are needed that regulate the interval between US exam and delivery to improve the predicting accuracy. Fifthly, the majority of pregnant women in our hospital did not undergo 3D ultrasound or MR examinations. So we were unable to compare DNN model based on US with models based on other examinations such as 3D-US or MR. Finally, the EHR data in this study did not contain data on the thickness of subcutaneous fat, dietary habits, paternal contributions, and placental position, which were previously shown to be associated with fetal birth weight [26, 28, 29]. Future prospective studies should address these parameters as well.

### Interpretation

The report indicates that apart from the US data, the maternal features with the greatest impact on EFW were pre-pregnancy maternal weight, maternal OGTT2H, and BMI. According to previous studies, gestational diabetes mellitus mothers are more likely to have overweight babies [30, 31]. Maternal obesity leads to multidirectional effects on fetal growth (low birth weight, fetal growth restriction, and macrosomia) [32]. It might be related to genetic factors [33] and metabolism status [32, 34] of the mother.

Multiparas have data of previous deliveries that can be used to optimize the model to predict subsequent birth weight. We found that adding data of previous deliveries can predict the fetal weight of the current pregnancy more accurately. Genetic background may play a role. Previous reports have shown that mothers who previously delivered SGA or LGA neonates would increase the risk of delivered SGA or LGA neonates this time [26, 27].

The proposed prediction model was more accurate compared to both Hadlock’s method and MLR in predicting the EFW and therefore have a potential for aiding decision making in fetal development monitoring. Future prospective studies and large multi-center cohorts are needed to validate this findings and evaluate the clinical impact of the DNN model. Also the study did not assess the generalizability of the DNN model to other populations, such as women with pre-existing medical conditions or different ethnicities. Future research should focus on refining the machine learning models, investigate the performance of the DNN model in different populations, providing long-term monitoring of EFW throughout pregnancy, and investigating those cases in which the model prediction was less accurate.

## Conclusion

In conclusion, proposed prediction model was significantly more accurate compared to both Hadlock’s method and MLR in predicting the EFW and therefore have a potential for aiding decision making in fetal development monitoring. Future prospective studies and large multi-center cohorts are needed to validate these findings and evaluate the clinical impact of the DNN model.

## Supplementary Information

**Additional file 1:** **Table S1. **Basic characteristics of the cohort data. **Table S2. **The RMSE (g) of different layers and nodes during pre-training. **Table S3. **The mean and 95% confidence interval of percentage error (PE), absolute percentage error (APE), absolute error (AE) , root-mean-square error (RMSE), and predicting accuracy of all methods. **Table S4. **Ten predictive features selected for the non-first delivery group and the entire cohort. **Table S5. **The mean and 95% confidence interval of percentage error (PE), absolute percentage error (APE), absolute error (AE) , root-mean-square error (RMSE), and predicting accuracy of DNN models of firstdelivery group, non-first delivery group and the entire cohort. **Table S6. **The mean and 95% confidence interval of percentage error (PE), absolute percentage error (APE), absolute error (AE) , root-mean-square error (RMSE), and predicting accuracy DNN models after feature selection. **Table S7. **The cut-off point, sensitivity when the specificity at 90% of all methods on macrosomia prediction. The AUC and its 95% confidence interval of all methods. **Table S8. **The mean and 95% confidence interval of percentage error (PE), absolute percentage error (APE), absolute error (AE) , root-mean-square error (RMSE), and predicting accuracy of DNN and MLR models with only 4 ultrasound features compare to Hedlock. **Figure S1. **The performance of DNN and MLR models with only 4 ultrasound features compare to Hedlock. The mean deviation (± 1 SD), mean absolute error (MAE), **B**) mean absolute percentage error (MAPE), **C**) and root-mean-square error (RMSE), **D**) and their 95% confidence interval of EFW in different methods.

## Data Availability

All data generated or analysed during this study are included in this published article.

## Abbreviations

EFW: Estimated fetal weight
DNN: Deep neural network
SGA: Small-for-gestational-age
IUGR: Intrauterine growth retardation
LGA: Large-for-gestational-age
ANN: Artificial neural network
MAPE: Mean absolute percentage error
MAE: Mean absolute error
RMSE: Root mean square error
