# Supplementary material for: Fetal weight estimation based on deep neural network: a retrospective observational study
Source: BMC Pregnancy Childbirth. 2023 Aug 2;23:560. doi: 10.1186/s12884-023-05819-8 (PMC10394792; doi:10.1186/s12884-023-05819-8)

**Supplemental Material**

**Table S1. Basic characteristics of the cohort data.**

|  | N | Mean | Std. Deviation | C.V% |
| --- | --- | --- | --- | --- |
| Birth weight (g) | 34824 | 3348.70 | 422.17 | 0.13 |
| Available at pregnancy initiation | | | | |
| Maternal age | 34824 | 31.08 | 3.97 | 0.13 |
| Gravidity | 34822 | 1.87 | 1.09 | 0.59 |
| Parity | 34824 | 1.32 | 0.49 | 0.37 |
| Maternal weight before pregnant (kg) | 34206 | 55.59 | 8.09 | 0.15 |
| Maternal height (cm) | 34491 | 162.11 | 4.97 | 0.03 |
| Body mass index | 34202 | 21.14 | 2.80 | 0.13 |
| Maximum birth weight of previous babies (g) | 9687 | 3365.56 | 447.30 | 0.13 |
| Mean birth weight of previous babies (g) | 9687 | 3360.28 | 447.58 | 0.13 |
| Macrosomia in previous babies | 9687 | 0.08 | 0.27 | 3.40 |
| In-vitro fertilization or not | 34824 | 0.11 | 0.31 | 2.80 |
| Gathered throughout pregnancy | | | | |
| Gestational age (weeks) | 34824 | 39.07 | 1.17 | 0.03 |
| Maternal weight before delivery (kg) | 28874 | 69.71 | 23.73 | 0.34 |
| Weight gain during pregnancy | 28593 | 14.14 | 22.48 | 1.59 |
| Interval between weights measurement and delivery (days) | 29014 | 1.24 | 1.37 | 1.10 |
| Systolic blood pressure | 33642 | 111.32 | 13.05 | 0.12 |
| Diastolic blood pressure | 33642 | 68.92 | 11.09 | 0.16 |
| Interval between BP measurement and delivery (days) | 27385 | 1.29 | 1.87 | 1.45 |
| Fundal height | 27151 | 34.60 | 1.72 | 0.05 |
| Oral glucose tolerance test 0h | 32122 | 4.31 | 0.42 | 0.10 |
| Oral glucose tolerance test 1H | 33803 | 4.22 | 0.40 | 0.10 |
| Oral glucose tolerance test 2H | 33803 | 4.22 | 0.40 | 0.10 |
| Triglyceride (early pregnancy) | 30063 | 1.30 | 0.53 | 0.41 |
| Cholesterol (early pregnancy) | 30063 | 4.57 | 0.74 | 0.16 |
| Glycosylated hemoglobin (early pregnancy) | 29582 | 5.12 | 0.30 | 0.06 |
| Glycosylated hemoglobin (middle pregnancy) | 30246 | 4.86 | 0.31 | 0.06 |
| Albumin (early pregnancy) | 29905 | 30.24 | 17.89 | 0.59 |
| Last ultrasound examination | | | | |
| Biparietal diameter | 34571 | 93.78 | 3.58 | 0.04 |
| Head circumference | 33316 | 319.37 | 20.67 | 0.06 |
| Femur length | 34824 | 68.96 | 4.39 | 0.06 |
| Humerus length | 34819 | 60.38 | 6.42 | 0.11 |
| Abdominal transverse diameter | 34824 | 101.25 | 7.84 | 0.08 |
| Abdominal anteroposterior diameter | 34824 | 102.92 | 6.87 | 0.07 |
| Amniotic fluid depth 1 | 34791 | 31.91 | 15.62 | 0.49 |
| Amniotic fluid depth 2 | 34795 | 29.78 | 15.66 | 0.53 |
| Amniotic fluid depth 3 | 34795 | 30.34 | 12.63 | 0.42 |
| Amniotic fluid depth 4 | 34791 | 31.08 | 12.51 | 0.40 |
| Total amniotic fluid index | 34778 | 123.88 | 81.93 | 0.66 |
| Placental thickness | 34812 | 36.10 | 6.23 | 0.17 |
| Umbilical artery AB | 34609 | 2.26 | 2.36 | 1.05 |
| Umbilical artery PI | 34613 | 0.83 | 2.39 | 2.88 |
| Umbilical artery RI | 34609 | 0.58 | 3.19 | 5.54 |
| Interval between ultrasound and delivery (days) | 34824 | 6.71 | 4.04 | 0.60 |

**Table S2. The RMSE (g) of different layers and nodes during pre-training.**

| **Layers** | **Nodes** | | | | | | | |
| --- | --- | --- | --- | --- | --- | --- | --- | --- |
|  | 10 | 15 | 20 | 25 | 30 | 35 | 40 | 45 |
| 1 | 247.91 | 246.00 | 248.66 | 250.33 | 246.10 | 246.46 | 249.09 | 247.15 |
| 2 | 242.04 | 241.46 | 241.59 | 242.00 | 240.98 | 241.56 | 243.02 | 243.32 |
| 3 | 245.02 | 241.03 | 242.76 | 242.97 | 240.36 | 242.83 | 241.26 | 241.55 |
| 4 | 246.18 | 243.45 | 249.09 | 242.95 | 244.20 | 247.65 | 244.23 | 242.80 |
| 5 | 245.16 | 249.07 | 247.60 | 242.10 | 245.18 | 244.50 | 246.94 | 243.81 |
| 6 | 257.36 | 248.82 | 248.02 | 245.89 | 246.38 | 245.66 | 247.65 | 246.42 |
| 7 | 250.23 | 253.04 | 256.66 | 249.20 | 247.14 | 250.26 | 249.87 | 250.01 |
| 8 | 255.40 | 257.91 | 251.25 | 249.21 | 248.84 | 245.76 | 249.23 | 247.32 |

**Table S3. The mean and 95% confidence interval of percentage error (PE), absolute percentage error (APE), absolute error (AE) , root-mean-square error (RMSE), and predicting accuracy of all methods.**

|  | PE | | APE | | AE (g) | | RMSE (g) | 95% CI | Accuracy (AE<250g) | Accuracy (APE<10%) |
| --- | --- | --- | --- | --- | --- | --- | --- | --- | --- | --- |
|  | Mean | 95% CI | Mean | 95% CI | Mean | 95% CI |  |  |  |  |
| Hedlock 1 | 0.55% | 0.42%-0.60% | 6.46% | 6.41%-6.52% | 214.95 | 213.48-217.10 | 271.35 | 269.69-274.15 | 64.93% | 78.60% |
| Hedlock 2 | 2.44% | 2.35%-2.53% | 6.84% | 6.78%-6.90% | 224.75 | 222.90-226.60 | 281.71 | 279.44-283.89 | 62.47% | 76.08% |
| MLR | 0.57% | 0.46%-0.62% | 5.90% | 5.81%-5.92% | 195.12 | 193.10-196.43 | 254.62 | 245.50-249.85 | 69.98% | 82.76% |
| DNN | 0.85% | 0.71%-0.87% | 5.79% | 5.70%-5.81% | 189.64 | 187.95-191.16 | 240.36 | 238.24-242.31 | 71.36% | 83.87% |

**Table S4.** Ten predictive features selected for the non-first delivery group and the entire cohort

|  | **Mean** | **Std. Deviation** |
| --- | --- | --- |
| **Entire Data** |  |  |
| Abdominal anteroposterior diameter | 101.25 | 7.84 |
| Abdominal transverse diameter | 102.92 | 6.87 |
| Humerus length | 60.38 | 6.42 |
| Femur length | 68.96 | 4.39 |
| Biparietal diameter | 93.78 | 3.58 |
| Head circumference | 319.37 | 20.67 |
| Gestational age (weeks) | 39.07 | 1.17 |
| Interval between ultrasound and delivery (days) | 6.71 | 4.04 |
| Weight before delivery | 69.71 | 23.73 |
| Fundal height | 34.60 | 1.72 |
| **Non-first Delivery** | | |
| Gestational age (weeks) | 38.78 | 1.23 |
| Weight before pregnant (kg) | 56.34 | 7.99 |
| Mean birth weight of previous babies (g) | 3364.94 | 445.94 |
| Maximum birth weight of previous babies (g) | 3359.66 | 446.22 |
| Abdominal transverse diameter (mm) | 101.43 | 6.69 |
| Abdominal anteroposterior diameter (mm) | 103.14 | 7.05 |
| Femur length (mm) | 68.76 | 2.95 |
| Biparietal diameter (mm) | 93.26 | 3.74 |
| Humerus length (mm) | 60.38 | 6.42 |
| Interval between ultrasound and delivery (days) | 6.64 | 3.95 |

**Table S5. The mean and 95% confidence interval of percentage error (PE), absolute percentage error (APE), absolute error (AE) , root-mean-square error (RMSE), and predicting accuracy of DNN models of first delivery group, non-first delivery group and the entire cohort.**

|  | PE (%) | | APE (%) | | AE (g) | | RMSE (g) | RMSE 95% CI | Accuracy (AE<250g) | Accuracy (APE<10%) |
| --- | --- | --- | --- | --- | --- | --- | --- | --- | --- | --- |
|  | Mean | 95% CI | Mean | 95% CI | Mean | 95% CI |  |  |  |  |
| First Delivery | 0.62% | 0.53%-0.72% | 5.82% | 5.76%-5.88% | 190.38 | 188.47-192.28 | 242.57 | 239.60-245.51 | 71.30% | 83.51% |
| Entire Data | 0.85% | 0.77%-0.93% | 5.79% | 5.73%-5.84% | 189.64 | 188.09-191.19 | 240.36 | 238.38-242.31 | 71.36% | 83.87% |
| Non-First Delivery | 0.53% | 0.38%-0.67% | 5.61% | 5.52%-5.71% | 186.87 | 183.87-289.87 | 239.9 | 234.06-245.59 | 72.26% | 85.07% |

**Table S6. The mean and 95% confidence interval of percentage error (PE), absolute percentage error (APE), absolute error (AE) , root-mean-square error (RMSE), and predicting accuracy DNN models after feature selection.**

|  | PE | | APE | | AE (g) | | RMSE (g) | RMSE 95% CI | Accuracy (AE<250g) | Accuracy (APE<10%) |
| --- | --- | --- | --- | --- | --- | --- | --- | --- | --- | --- |
|  | Mean | 95% CI | Mean | 95% CI | Mean | 95% CI |  |  |  |  |
| DNN Entire Data After Feature Selection | 0.60% | 0.52%-0.69% | 5.81% | 5.75%-5.86% | 192.21 | 190.54-193.87 | 243.8 | 241.66-245.91 | 70.43% | 83.52% |
| DNN Non-first Delivery After Feature Selection | -0.75% | -0.0028 | 5.59% | 5.55%-5.68% | 189.75 | 186.76-192.74 | 241.98 | 238.13-245.77 | 71.61% | 84.98% |

**Table S7. The cut-off point, sensitivity when the specificity at 90% of all methods on macrosomia prediction. The AUC and its 95% confidence interval of all methods.**

|  | **Specificity = 90%** | | **Area under the curve** | **95% CI of AUC** | |
| --- | --- | --- | --- | --- | --- |
|  | **cut-off point** | **sensitivity** |  | **Lower bound** | **Upper bound** |
| **Hedlock’s formula 1** | **3830.50** | **0.658** | **0.894** | **0.888** | **0.901** |
| **Hedlock’s formula 2** | **3830.50** | **0.658** | **0.895** | **0.888** | **0.901** |
| **MLR model** | **3679.45** | **0.712** | **0.914** | **0.908** | **0.919** |
| **DNN model** | **3680.80** | **0.738** | **0.921** | **0.916** | **0.926** |

**Table S8. The mean and 95% confidence interval of percentage error (PE), absolute percentage error (APE), absolute error (AE) , root-mean-square error (RMSE), and predicting accuracy of DNN and MLR models with only 4 ultrasound features compare to Hedlock.**

|  | PE | | APE | | AE (g) | | RMSE (g) | 95% CI | Accuracy (AE<250g) | Accuracy (APE<10%) |
| --- | --- | --- | --- | --- | --- | --- | --- | --- | --- | --- |
|  | Mean | 95% CI | Mean | 95% CI | Mean | 95% CI |  |  |  |  |
| Hedlock 1 | 0.55% | 0.42%-0.60% | 6.46% | 6.41%-6.52% | 214.95 | 213.48-217.10 | 271.35 | 269.69-274.15 | 64.93% | 78.60% |
| Hedlock 2 | 2.44% | 2.35%-2.53% | 6.84% | 6.78%-6.90% | 224.75 | 222.90-226.60 | 281.71 | 279.44-283.89 | 62.47% | 76.08% |
| MLR | 0.94% | 0.69%-0.87% | 6.56% | 6.42%-6.53% | 215.79 | 212.37-216.00 | 312.41 | 268.11-275.02 | 65.12% | 78.65% |
| DNN | 0.95% | 0.73%-0.90% | 6.41% | 6.30%-6.41% | 210.23 | 207.99-211.50 | 265.48 | 262.40-266.78 | 66.13% | 79.47% |

**Figure S1. The performance of DNN and MLR models with only 4 ultrasound features compare to Hedlock. The mean deviation (± 1 SD), mean absolute error (MAE), B) mean absolute percentage error (MAPE), C) and root-mean-square error (RMSE), D) and their 95% confidence interval of EFW in different methods.**


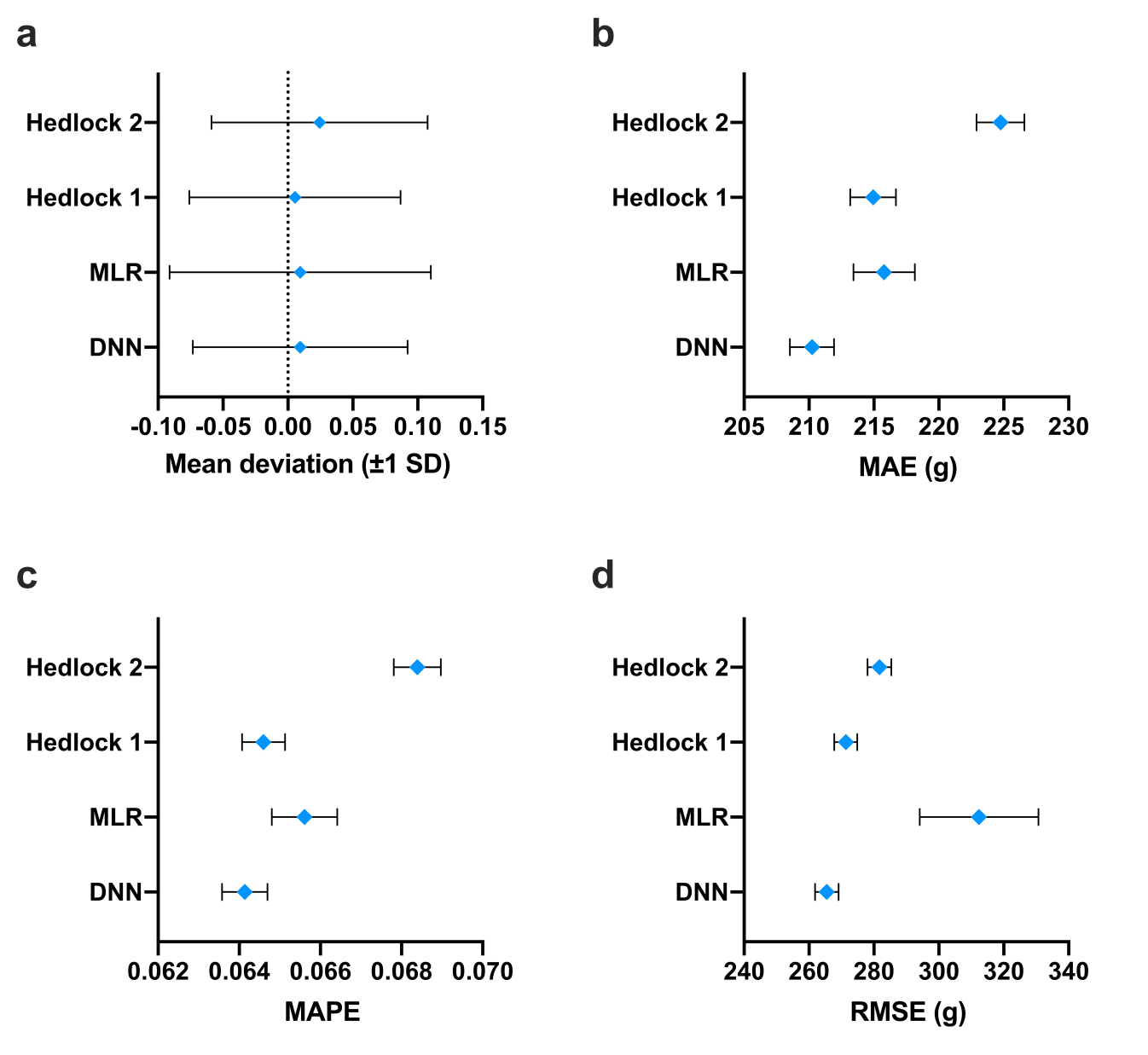

Supplement: Supplementary file 1 — Additional file 1: Table S1. Basic characteristics of the cohort data. Table S2. The RMSE (g) of different layers and nodes during pre-training. Table S3. The mean and 95% confidence interval of percentage error (PE), absolute percentage error (APE), absolute error (AE) , root-mean-square error (RMSE), and predicting accuracy of all methods. Table S4. Ten predictive features selected for the non-first delivery group and the entire cohort. Table S5. The mean and 95% confidence interval of percentage error (PE), absolute percentage error (APE), absolute error (AE) , root-mean-square error (RMSE), and predicting accuracy of DNN models of firstdelivery group, non-first delivery group and the entire cohort. Table S6. The mean and 95% confidence interval of percentage error (PE), absolute percentage error (APE), absolute error (AE) , root-mean-square error (RMSE), and predicting accuracy DNN models after feature selection. Table S7. The cut-off point, sensitivity when the specificity at 90% of all methods on macrosomia prediction. The AUC and its 95% confidence interval of all methods. Table S8. The mean and 95% confidence interval of percentage error (PE), absolute percentage error (APE), absolute error (AE) , root-mean-square error (RMSE), and predicting accuracy of DNN and MLR models with only 4 ultrasound features compare to Hedlock. Figure S1. The performance of DNN and MLR models with only 4 ultrasound features compare to Hedlock. The mean deviation (± 1 SD), mean absolute error (MAE), B) mean absolute percentage error (MAPE), C) and root-mean-square error (RMSE), D) and their 95% confidence interval of EFW in different methods. [file 12884_2023_5819_MOESM1_ESM.docx]
